# An Integrated Approach for the Valorization of Cheese Whey

**DOI:** 10.3390/foods10030564

**Published:** 2021-03-09

**Authors:** Francisco J. Barba

**Affiliations:** Nutrition and Bromatology Area, Department of Preventive Medicine and Public Health, Food Science, Toxicology and Forensic Medicine, Faculty of Pharmacy, Universitat de València, Avda. Vicent Andrés Estellés, s/n 46100 Burjassot, València, Spain; francisco.barba@uv.es; Tel.: +34-96-3544-972; Fax: +34-96-3544-954

**Keywords:** cheese, whey, valorization, innovative approaches, biorefining

## Abstract

Taking into account the large amount of whey that is produced during the cheese production process and the constant demand by society for more sustainable processes, in accordance with Sustainable Development Goals (SDGs) and the circular economy concept, it is necessary to adapt two-unit operations into a single process, allowing us to not only valorize a part of the whey but the whole process, which is known as bioprocess integration. In this sense, the adaptation of different processes, for example, physicochemical (micro, ultra and nanofiltration) and fermentation, that are commonly used to obtain proteins, lactose and other compounds with different activities (antioxidant, antifungal, etc.) could be integrated to achieve a complete recovery of the cheese whey. Likewise, keeping in mind that one of the main drawbacks of cheese whey is the great microbial load, some innovative processing technologies, such as high hydrostatic pressures, electrotechnologies and ultrasound, can allow both the development of new foods from whey as well as the improvement of the nutritional and organoleptic properties of the final products prepared with cheese, and thus reducing the microbial load and obtaining a safe product could be incorporated in the cheese whey valorization process.

## 1. Introduction

Due to the recent efforts to increase sustainability at the agri-food level and in full correspondence with both the Sustainable Development Goals (SDGs) and the circular economy concept, there has been an increased growth in the interest to valorize the resources obtained during different food production processes [1,2].

Whey is the main by-product obtained by the dairy industry, representing a global whey production of ~200 million tonnes (MT) [3,4,5], with ~40 MT being produced in the European Union [6]. Although ≈50% of residual whey is being valorized as a source of high-added-value compounds for the food or pharmaceutical industries, mainly proteins of high biological value, lactose, lactic acid and minerals, there is still a high proportion of this whey that is wasted, thus promoting environmental pollution due to its high biological oxygen demand and important organic load [7,8].

**Figure 1:**
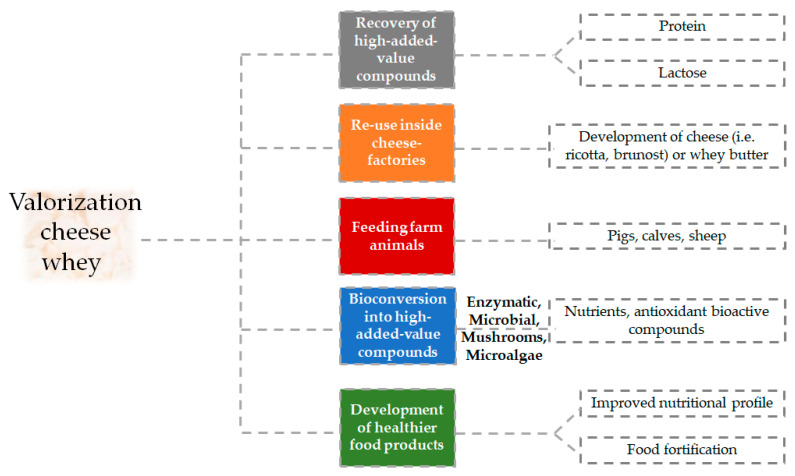
Conventional and emerging applications of cheese whey.

Thus, different research groups have studied potential strategies to valorize cheese whey. A schematic representation of some of the different conventional and emerging applications of whey at the food level is shown in Figure 1.

As it is stated in the figure, cheese whey can be re-used inside the cheese factories to produce other fresh products such as, for example, cheese such as ricotta or brunost, and whey butter. Moreover, it has traditionally been used for feeding animals such as pigs, but it is also used after specific processing for the feeding of calves and sheep [6,7,9].

Cheese whey can also be directly used as a food ingredient or concentrated for protein powders [7]. In this sense, 50% of cheese whey production is used for the development of food and feed products, with half of this amount being utilized directly in liquid form, 30% as powdered cheese whey, 15% as lactose and side streams and the remaining amount as protein concentrates [6,10].

## 2. Whey Protein Recovery

Different physicochemical processes have been used to obtain protein-rich concentrates from cheese whey, including filtration processes such as microfiltration, ultrafiltration and nanofiltration. However, despite the high quality of the protein extracts obtained, the deproteinized part still presents contamination due to the important lactose content (from 3.60 to 3.95%) [11,12]. More recently, other authors have evaluated the development of native whey, which is of great interest from the point of view of infant nutrition and as a functional food ingredient, produced by the microfiltration of skim milk, and they have studied the effect of different processes to obtain whey powder, such as standard thermal pasteurization, membrane concentration and spray drying, to see how these processes affect the quality of proteins, observing that pasteurization keeps the proteins present in the native unchanged whey [13]. As mentioned before, the whey obtained after milk microfiltration is known as native whey. It has different nutritional and technological advantages compared to the whey obtained after cheese production. Among these advantages, the greater release of β-casein should be highlighted, as well as the processing at low temperatures, thus avoiding protein denaturation [13].

Whey proteins have been shown to promote the activity of glutathione peroxidase, a key enzyme in the fight against oxidative stress [14]. Furthermore, lactoferrin represents between 1 and 2% of the total proteins present in whey [15], having a great antioxidant capacity and being able to scavenge free radicals due to its sulfur-containing amino acids in its structure and the chelation of transition metals [16,17], as well as its important antifungal activity [18].

Other studies utilized cheese whey as a healthy alternative source of protein [4]. For instance, whey proteins comprise approximately 20% of total bovine milk proteins, thus being a great and balanced source of essential amino acids [19]. In this sense, some authors have used cheese whey to increase the nutritional value of the final food products [20]. For example, adding whey proteins into breads that lack essential amino acids, lysine, tryptophan and methionine can be a good strategy to improve the nutritional profile of the new developed food products. Moreover, whey protein concentrates improve the sensory quality of bread [21].

In addition, in recent studies, different researchers have investigated the addition of whey as a meat protein replacer in order to develop healthier meat products and evaluated its impact on the nutritional composition (moisture, fat, ash, carbohydrates, proteins and amino acid profile) and physicochemical properties (color, hardness, adhesiveness, elasticity, gumminess and chewiness) of the final meat products, obtaining interesting results from a nutritional and physicochemical point of view [22].

## 3. Development of Preservative Compounds

Furthermore, it is also possible to obtain compounds with antioxidant and/or antifungal activity from cheese whey proteins through enzymatic transformation or fermentation by different common bacterial, yeast or fungal species. For instance, whey proteins are a source of bioactive peptides, which are released during protein hydrolysis induced mostly by enzymatic or fermentation processes. These peptides have demonstrated in vitro antioxidant activity, in addition to possessing other biologically interesting characteristics such as antioxidant, antibacterial, antidiabetic, antihypertensive or angiotensin converting enzyme (ACE)-inhibition, antithrombotic, immunomodulatory, anticancer, mineral binding, opioid, and satiating properties [23]. Moreover, some authors also observed how the addition of whey protein bioactive peptides showed promising results during the storage of pork under refrigeration, both reducing oxidative degradation and reducing cooking loss [24].

## 4. Whey Bioconversion

Whey acts as a support for the growth of bacteria, mushrooms, and microalgae, among others, thus acting as a substrate for the production of high-added-value compounds such as polysaccharides, carotenoids or chlorophyll, among other compounds [7,25]. In fact, it is possible to use fermented whey to not only fortify some foods, but also to improve their shelf life. For example, some authors supplemented poultry feed with fermented whey and they observed an enhancement (2–4-fold) of the antifungal activity compared to the control feed [26]. In another study, it was observed how the use of lactic acid fermented whey powder led to the slight inactivation (0.5–0.6 log cfu/g) of *P. expansum*, a toxigenic fungus in loaves of bread [27].

Few selected strains grow fast on whey as a medium and exhibit higher antioxidant activity. Lactic acid bacteria mostly carry out proteolysis in whey, and a higher degree of proteolysis relates to the DPPH or ABTS antioxidant potential [28]. Therefore, the fermentation of whey provides immense potential of waste valorization to generate bioactives and proficient alternatives for the shelf-life enhancement of fortified food products.

Likewise, cheese whey is a source of fatty acids, which also have an interesting antifungal action, especially those free fatty acids obtained after saponification, which are able to inhibit the growth of *Aspergillus fumigatus* and *C. albicans*, with γ-linolenic acid being the most potent antifungal compound [29].

## 5. Whey Lactose Valorization

**Figure 2:**
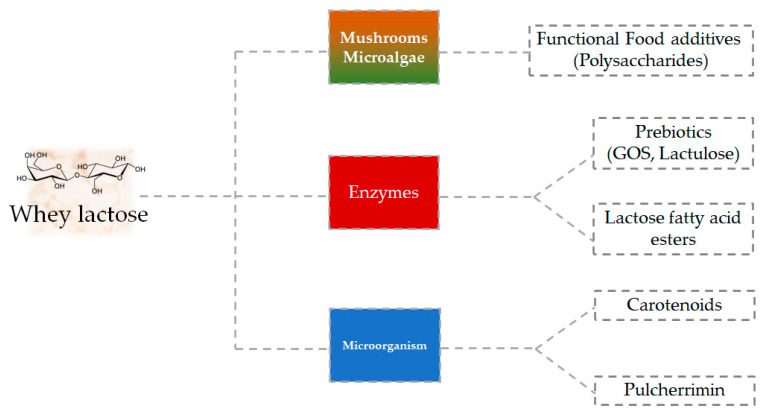
Innovative strategies for valorization of lactose from whey cheese. GOS: Galacto-oligosaccharides.

In addition to the aforementioned valorization routes, in recent years the use of lactose from whey as a substrate for the production (enzymatic, microbial, mushrooms or microalgae) of prebiotics (e.g., oligosaccharides and lactulose), lactose fatty acid esters, carotenoids, and pulcherrimin, as well as the development of some polysaccharides that can be used as functional food additives, has attracted a growing interest from both academics and industries (food, pharmaceutical, cosmetic, etc.) (Figure 2) [7]. The fermentative way of processing cheese whey also allows us to obtain biofuels and bioplastics, which differ according to the type of microorganism inoculated [7,25].

It should also be noted that the specific physicochemical characteristics of the proteins allow us to obtain products with highly acceptable structural and rheological characteristics compared to other compounds. In addition, the proteins present in cheese whey allow us to obtain macro-, micro-, and nano-structures with numerous promising food applications, such as vehicle carrying for various bio-compounds, flavors, or nutrients. For example, whey proteins from cheese have been used for the development of edible films and coatings as well as whey protein hydrogels [7]. In this sense, whey protein hydrogels are considered to be a useful tool for the preservation of probiotic bacteria after the application of a heat treatment, as well as an interesting vehicle for the controlled release of nutraceuticals. On the other hand, edible films and coatings are used both for preservation purposes (i.e., antimicrobial component delivery, antioxidant activity, UV light protection, etc.) and to improve the mechanical and thermal properties. Moreover, they can be also used as microencapsulating agents to improve the viability of probiotic microorganisms, as well as vehicles to allow the release and controlled solubilization of bioactive compounds [7].

## 6. Author Viewpoint

Taking into account that there are different approaches for cheese whey valorization but none are definitive, it is necessary to incorporate more two-unit operations in a single process, which is known as a bioprocess integration. In this sense, keeping in mind the conventional and emerging routes for the valorization of cheese whey, its re-use inside the cheese factories to produce other fresh food products (i.e., cheese or whey butter), as well as to feed farm animals, and the recovery of proteins (i.e., whey protein concentrates) and lactose are well established. However, there is a need to integrate all the processes in a multistep biorefining strategy which can allow the whole valorization of cheese whey. For example, those side streams lacking an effective disposal such as whey permeate and delactosed whey permeate could be used to develop cost competitive bio-based products or to configure bioprocesses for multiple end-products [7]. So, a cascade approach could be established for a complete cheese whey valorization (zero waste), including the development or recovery of high-added-value compounds (nutrients, antioxidant bioactive compounds) through biotransformation or conventional or innovative extraction techniques, thus allowing their use as food additives, nutraceuticals, etc., or allowing them to be incorporated into new foods (i.e., food fortification, supplementation, etc.). After that, the remaining product of cheese whey valorization is still an interesting tool to develop new materials such as hydrogels, biofuels, bioplastics, etc.

To achieve this last and primary objective, taking into account that one of the main drawbacks when it comes to valorizing cheese whey is the high microbial load, innovative processing technologies, such as high hydrostatic pressures, electrotechnologies, ultrasound, etc., could be a potential strategy to be used. These technologies, apart from reducing the microbial load using mild temperatures, could be used to develop tailor made processes for the production of new food products/compounds [30,31,32].

## Data Availability

Not applicable.
